# Cold Resection for Superficial Non‐Ampullary Duodenal Epithelial Tumors: Current Evidence and Clinical Perspectives

**DOI:** 10.1002/deo2.70429

**Published:** 2026-09-07

**Authors:** Yuichiro Mikuriya, Kohei Takizawa, Nobuhisa Minakata, Kazuo Shiotsuki

**Affiliations:** ^1^ Department of Gastroenterology Kanagawa Cancer Center Kanagawa Japan

**Keywords:** adenoma, duodenal neoplasms, endoscopic mucosal resection, narrow band imaging, treatment outcome

## Abstract

Superficial non‐ampullary duodenal epithelial tumors (SNADETs) are increasingly treated with endoscopic resection. Although conventional thermal techniques are effective, the thin duodenal wall and distinctive anatomy have driven interest in cold resection techniques that avoid electrocautery‐related injury. This narrative review individually appraises cold snare polypectomy (CSP), cold forceps polypectomy (CFP), and cold snare endoscopic mucosal resection (CS‐EMR) for duodenal SNADETs. Management of selected small low‐grade adenomas should be individualized, with careful surveillance considered as an alternative to immediate resection. When resection is chosen, CSP is the principal cold resection technique for lesions <10 mm that are optically diagnosed as low‐grade adenomas using white‐light imaging and narrow‐band imaging. CSP appears technically feasible and safe for selected small SNADETs. However, its shallow resection depth limits vertical margin assessment. Available follow‐up data suggest low recurrence rates, and recurrent lesions are generally small and endoscopically manageable. This review also discusses the importance of pretreatment optical diagnosis, appropriate lesion selection, technical considerations, and structured surveillance after cold resection. CFP may be considered for carefully selected diminutive lesions, although the supporting evidence remains limited. Although CS‐EMR can enable resection of larger lesions, substantial recurrence rates and the reported risk of perforation argue against its routine use. Overall, CSP is the principal cold resection technique for selected small low‐grade adenomas, whereas CFP and CS‐EMR have more limited roles.

## 1 Introduction

Superficial non‐ampullary duodenal epithelial tumors (SNADETs) are rare, with a reported prevalence of 0.03%–0.40% [1, 2]. Their detection has increased with the widespread adoption of high‐definition endoscopy and image‐enhanced techniques, including narrow‐band imaging (NBI) [3]. The adenoma–carcinoma sequence has also been documented in the duodenum, supporting the importance of early detection and appropriate management of SNADETs [4].

For non‐invasive SNADETs, endoscopic resection has become an important alternative to surgery, supported by accumulating evidence of its safety and efficacy [5, 6, 7]. A recent analysis of the Japanese national cancer registry (2016–2020) reported a crude incidence of non‐ampullary duodenal cancer of 21.8–23.5 per 1,000,000 person‐years, with localized disease in 53.7% of patients. Endoscopic resection was the second most common treatment overall (26.7%), after surgery (32.0%), reflecting its important role in current clinical practice [8].

Selection of endoscopic techniques is guided by lesion size, morphology, location, and operator expertise [6]. Conventional thermal approaches, including endoscopic mucosal resection (EMR), underwater EMR (UEMR), and endoscopic submucosal dissection (ESD), remain established treatment options for SNADETs [5, 9]. Nevertheless, these methods are associated with relatively high adverse‐event rates in the duodenum because of its thin wall and exposure to bile and pancreatic juice [7].

In response to these safety concerns, cold resection techniques that avoid electrocautery have attracted growing interest because of their potential to reduce electrocautery‐related adverse events [5, 9, 10, 11, 12].

This narrative review provides an updated synthesis of the evidence on cold resection for SNADETs. Cold snare polypectomy (CSP), cold forceps polypectomy (CFP), and cold snare endoscopic mucosal resection (CS‐EMR) are appraised individually to inform technique selection. The review also integrates recent evidence on optical diagnosis and lesion selection and proposes a practical treatment framework centered on carefully selected small low‐grade adenomas.

## 2 Malignant Risk and Necessity of Treatment

In the World Health Organization classification, non‐ampullary duodenal epithelial tumors are classified along a continuum from adenoma (dysplasia/intraepithelial neoplasia) to adenocarcinoma [13]. Although SNADETs can progress along the adenoma–carcinoma pathway, evidence regarding the malignant transformation of low‐grade adenomas remains limited.

In a surveillance cohort, 20.9% of lesions initially diagnosed as low‐grade dysplasia progressed to high‐grade dysplasia (HGD), including 4.7% that eventually progressed to non‐invasive carcinoma; however, most lesions (79.1%) remained unchanged during follow‐up [4]. In another observational cohort of 86 SNADETs managed without immediate resection, macroscopic progression occurred in eight cases (9.3%). Final histology revealed adenocarcinoma in four of these cases, three of which showed submucosal invasion [14].

Additional observational series have reported lateral growth and occasional transformation to mucosal carcinoma during endoscopic follow‐up [15]. Although the European Society of Gastrointestinal Endoscopy (ESGE) guideline recommends considering endoscopic resection for all duodenal adenomas, the optimal management of small low‐grade adenomas remains uncertain [6]. Larger lesions (≥10 mm) are associated with a higher risk of progression, whereas small lesions (<5 mm) without high‐risk features may remain stable over time [16]. Because the supporting evidence is largely retrospective, the Japanese clinical practice guidelines only weakly recommend endoscopic treatment of non‐ampullary duodenal adenomas [17]. Management of small low‐grade adenomas should therefore be individualized, with careful endoscopic surveillance and endoscopic resection considered on a case‐by‐case basis [15, 16].

## 3 Duodenum‐Specific Challenges and Adverse Event Mechanisms

Once resection is deemed appropriate, the duodenum presents distinct technical challenges because its narrow, angulated lumen restricts endoscopic maneuverability [5, 9]. Moreover, its thin wall and exposure to bile and pancreatic juice increase the risk of delayed adverse events after electrocautery‐based resection [7].

These characteristics are reflected in the adverse‐event profile of thermal resection techniques. In a large multicenter analysis of 3,107 patients from 18 Japanese institutions, delayed adverse events occurred in 2.8%, 2.2%, and 6.8% after EMR, UEMR, and ESD, respectively, whereas delayed perforation occurred in 0.2% after both EMR and UEMR and in 2.3% after ESD [7]. Moreover, individual cohort studies have reported delayed bleeding rates of up to 17.5% and delayed perforation rates of 1–6.3% after thermal resection [18, 19, 20, 21]. Conversely, cold polypectomy (including CSP and CFP) was associated with a delayed adverse‐event rate of only 0.5% in the multicenter analysis by Kato et al. [7]. These findings support cold resection as a safety‐oriented approach for selected duodenal lesions.

## 4 The Concept of Cold Resection

Because the duodenum is a high‐risk site for endoscopic complications, thermal resection may not always be the optimal first‐line approach for small, benign‐appearing adenomas [5, 6]. Treatment selection should therefore balance procedural safety against long‐term local control [6]. Cold resection is defined as mechanical tissue removal without high‐frequency electrocautery [10]. By avoiding electrocautery, this approach may reduce deep thermal injury, although the resection depth is limited. For clarity, we first define the specific cold resection techniques addressed in this review.

As terminology varies across published studies, we apply the following definitions consistently throughout this review. CSP is performed without submucosal injection. CS‐EMR refers to cold snare resection after submucosal injection. CFP denotes mechanical removal with biopsy forceps. In this review, we consider the term “cold EMR,” as used in some prior studies, equivalent to CS‐EMR, regardless of the terminology used.

## 5 Role of Optical Diagnosis

Differentiating low‐grade adenoma from HGD or carcinoma before treatment is essential for selecting an appropriate endoscopic technique. Forceps biopsy has limited accuracy for detecting HGD or carcinoma, and histopathologic discrepancies between forceps biopsy and resection specimens are well documented in SNADETs [22, 23, 24]. Moreover, preoperative biopsy may induce submucosal fibrosis in the duodenum, making subsequent endoscopic resection more difficult [22].

White‐light imaging (WLI) and image‐enhanced endoscopy, including NBI, are recommended for distinguishing low‐grade adenomas from lesions with HGD or carcinoma [6, 25, 26]. On WLI, Vienna classification category 3 lesions typically appear as small, isochromatic or whitish, flat‐elevated lesions with uniform nodularity; a validated scoring system can assist in differentiating adenoma from HGD or carcinoma [27].

Small lesion size alone does not exclude HGD or carcinoma. Although the frequency of Vienna classification category 4 or 5 lesions increases with tumor size, these lesions have also been reported in small SNADETs [28]. C4/C5 lesions reportedly accounted for 37% of SNADETs measuring 1–9 mm [25], and the proportion of HGD or carcinoma among diminutive lesions ≤5 mm has ranged from 35.7% to 60.2% in selected cohorts [29, 30]. Therefore, careful optical assessment is essential before selecting cold resection, even for lesions ≤10 mm [3, 25, 29, 30]. Several endoscopic features have been reported to predict HGD or carcinoma. On WLI, reddish coloration, depressed or mixed‐type morphology, and heterogeneous or absent nodularity suggest advanced histology [27, 29, 30]. Magnifying NBI may further support risk stratification because mixed or irregular surface patterns, irregular vessel patterns, absent or disappeared surface patterns, and unclassified vascular patterns have been associated with HGD or carcinoma [3, 25, 28, 31]. In addition, the distribution of white opaque substance (WOS) and milk‐white mucosa (MWM) provides useful diagnostic information. Negative WOS or limited MWM distribution, including marginal or non‐entire MWM, suggests Vienna classification category 4/5 lesions [32, 33, 34]. Conversely, homogeneous or extensive WOS/MWM supports a diagnosis of low‐grade adenoma [33, 34]. Therefore, even for lesions <10 mm, reddish coloration, depression, irregular surface architecture, negative WOS, or abnormal MWM distribution should prompt caution against CFP or CSP. In such cases, a resection method that permits more reliable histologic assessment should be considered. When the optical diagnosis strongly suggests a low‐grade adenoma, cold resection can serve as both a diagnostic and therapeutic procedure, minimizing procedural risk while avoiding the limitations of preoperative biopsy.

## 6 Clinical Outcomes and Efficacy of Cold Resection for SNADETs

### 6.1 Indications and Technique Selection

Because cold resection is mechanically limited in depth, its indication depends on tumor invasiveness [35]. Pathologic studies have demonstrated that CSP tends to produce a shallower resection plane than UEMR and may not reliably include the submucosa, thereby limiting vertical margin adequacy and histologic staging when advanced pathology is unexpectedly present [35, 36]. Therefore, cold resection should be avoided for lesions with suspected HGD, carcinoma, or submucosal invasion. For such lesions, techniques that permit reliable vertical margin assessment, such as UEMR, EMR, or ESD, are preferred [6, 17, 19, 35, 36].

For lesions optically diagnosed as low‐grade adenomas, technique selection is guided primarily by size. Standard CSP is appropriate only when both criteria are met: (i) lesion size <10 mm and (ii) an optical diagnosis of low‐grade adenoma (Vienna classification category 3) based on careful endoscopic assessment [10, 25, 36].

For diminutive lesions (≤6 mm), CFP may be considered a minimally invasive option. However, current evidence is limited to a single small cohort; therefore, its use should be restricted to carefully selected lesions within a structured follow‐up strategy [37].

CS‐EMR has been evaluated for larger benign‐appearing lesions that are difficult to remove with standard CSP. Although this technique may broaden the indications for cold resection, recurrence remains relatively frequent, and perforation has been reported [38, 39, 40]. For intermediate‐sized non‐ampullary duodenal adenomas, UEMR may be preferable when reliable resection is required. In a multicenter prospective study of lesions ≤20 mm, UEMR achieved a non‐recurrence rate of 97.2%, with delayed bleeding in 1.2% and no perforation [41]. Therefore, current evidence does not support the routine use of CS‐EMR.

**FIGURE 1:**
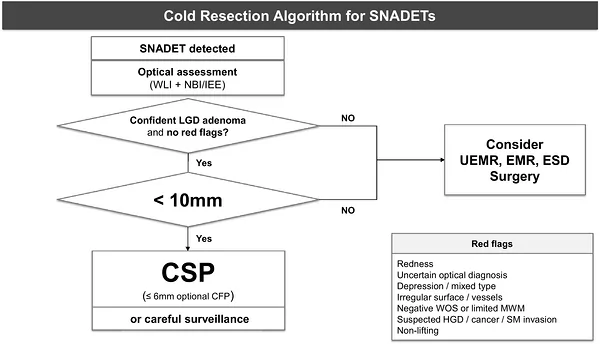
Clinical decision algorithm for cold resection of superficial non‐ampullary duodenal epithelial tumors. CSP is the principal cold resection technique for carefully selected lesions <10 mm that are optically consistent with low‐grade adenoma and have no red flags for high‐grade dysplasia, carcinoma, or submucosal invasion. CFP may be considered for selected diminutive lesions ≤6 mm. Lesions with an uncertain optical diagnosis, red flags, or a size ≥10 mm should generally be managed using techniques that permit more reliable histologic assessment, such as UEMR, EMR, ESD, or surgery, as appropriate. CS‐EMR is not shown because current evidence does not support its routine use. CFP, cold forceps polypectomy; CSP, cold snare polypectomy; CS‐EMR, cold snare endoscopic mucosal resection; EMR, endoscopic mucosal resection; ESD, endoscopic submucosal dissection; HGD, high‐grade dysplasia; IEE, image‐enhanced endoscopy; LGD, low‐grade dysplasia; MWM, milk‐white mucosa; NBI, narrow‐band imaging; SM, submucosal; SNADETs, superficial non‐ampullary duodenal epithelial tumors; UEMR, underwater endoscopic mucosal resection; WLI, white‐light imaging; WOS, white opaque substance.

Overall, CSP is the principal cold resection technique for small, non‐invasive adenomas. Technique selection is guided primarily by lesion size and optical diagnosis (Figure 1).

The key clinical outcomes of cold resection techniques for duodenal SNADETs, including technical success, histologic outcomes, adverse events, and recurrence, are summarized in Table 1.

**TABLE 1:** Summary of clinical studies evaluating cold resection techniques for superficial non ampullary duodenal epithelial tumors.

Technique	Study/design	Lesions	Technical outcome	Histology/margins	Safety	Recurrence/follow‐up
CSP	Takizawa 2022 [10], prospective pilot	21 lesions, ≤10 mm	CSP completion 86%; en bloc 81%	HM−/HM+/HMX 9/1/11; VM negative	Delayed bleeding 0%; perforation 0%	5.6% at 3 months
CSP/CFP	Maruoka 2017 [12], prospective	CSP 30; CFP 9, ≤6 mm	En bloc: CSP 96.7%; CFP 77.8%	R0 58.8% overall; CSP 17/25; CFP 3/9	Delayed bleeding 0%; perforation 0%	0% at 3 months
CSP	Okimoto 2022 [42], retrospective	46 lesions, ≤7 mm	En bloc 97.8%	R0 70.3%; horizontal margins often indeterminate	Delayed bleeding 0%; perforation 0%	2.7% at 12 months; median FU 48 months
CSP	Miyazaki 2024 [36], RCT	65 lesions, ≤12 mm	En bloc 95.4%	R0 61.5%; SVR0 41.5%	Delayed bleeding 1.5%; perforation 0%	1.5% at 6 months
CFP	Kanzaki 2022 [37], multicenter prospective	39 lesions, ≤6 mm	Single CFP disappearance 89.7%	LGD only	Delayed bleeding 0%; perforation 0%	12‐month disappearance 97.4%
CS‐EMR	Wilson 2024 [40], multicenter retrospective	127 lesions, ≥1 cm	Technical success 96.9%; piecemeal 81.1%	NR	AEs 3.9%; perforation 0%	31.5%; median FU 187 days
CS‐EMR	Wang 2023 [38], comparative	50 lesions, ≥15 mm	Technical success 100%; piecemeal 100%	NR	IPB 2.0%; PPB 4.0%; immediate perforation 4.0%	24.4% vs. 2.3% with EMR‐T

Abbreviations: AEs, adverse events; CFP, cold forceps polypectomy; CSP, cold snare polypectomy; CS‐EMR, cold snare endoscopic mucosal resection; EMR‐T, thermal endoscopic mucosal resection; FU, follow‐up; HM, horizontal margin; HMX, indeterminate horizontal margin; IPB, intraprocedural bleeding; LGD, low‐grade dysplasia; NR, not reported; PPB, postprocedural bleeding; R0, complete resection with negative margins; SVR0, sufficient vertical R0; VM, vertical margin.

## 7 Technical Feasibility and Resection Outcomes

### 7.1 Cold Snare Polypectomy

Among duodenal CSP series focusing on diminutive and small lesions, en bloc resection rates have ranged from approximately 81% to 97.8%, and technical success (completion without conversion) has been reported in 86%–100% of cases [10, 12, 42]. By contrast, histologic R0 resection rates have been more modest, ranging from approximately 68% to 70.3% in observational series [12, 42].

A randomized controlled trial (RCT) of 130 patients that compared UEMR with CSP for lesions ≤12 mm reported histologic R0 rates of 61.5% and 70.3% for CSP and UEMR, respectively. The sufficient vertical R0 (SVR0) rate was also lower with CSP than with UEMR (41.5% vs. 65.6%) [36]. These findings likely reflect frequent indeterminate horizontal margins and the limited depth of CSP resection [12, 35, 36, 42]. In a pathology‐based comparison, CSP reached the submucosa in only 21.8% of cases, indicating that specimens are often confined to the mucosa or muscularis mucosae [35].

From a clinical perspective, this shallower resection may be acceptable when the optical diagnosis confidently supports a low‐grade adenoma with expected mucosal confinement [10, 25, 36]. CSP may serve as an excisional diagnostic approach, offering a higher diagnostic yield than forceps biopsy while minimizing thermal injury [10, 22]. Nonetheless, when endoscopic features suggest HGD or carcinoma, techniques that permit more reliable vertical margin assessment should be selected [6, 36]. Overall, CSP is technically feasible for small SNADETs, although its inherent limitations for histopathologic staging should be recognized [35, 36].

### 7.2 Cold Forceps Polypectomy

For diminutive lesions, CFP may be considered a minimally invasive option. In a multicenter prospective study of 39 low‐grade adenomas ≤6 mm, CFP achieved a 12‐month disappearance rate of 97.4%, suggesting potential usefulness in carefully selected diminutive lesions within a structured surveillance strategy [37]. Nevertheless, this evidence comes from a single study with a small cohort. Further prospective data are needed before broader adoption can be recommended. Currently, CFP should be considered a limited option for carefully selected diminutive lesions rather than a standard approach.

### 7.3 Cold Snare‐EMR

For larger lesions that are difficult to capture with standard CSP, CS‐EMR has been investigated. In a meta‐analysis of six studies (178 lesions), pooled technical success was 95.8%, whereas pooled recurrence was 21.2% [39]. A multicenter study of 127 adenomas ≥1 cm reported a recurrence rate of 31.5% [40]. In a comparative study of large adenomas (≥15 mm; median, 30 mm), CS‐EMR was associated with a higher recurrence rate at first surveillance than conventional EMR (24.4% vs. 2.3%) [38]. These findings suggest that CS‐EMR is technically feasible, but recurrence remains an important limitation.

## 8 Safety Profile and Adverse Events

The principal advantage of cold resection is its favorable safety profile, largely attributable to the avoidance of thermal injury [10, 12, 35]. However, adverse event rates should be interpreted cautiously because adverse‐event definitions and management practices vary across studies.

### 8.1 Cold Snare Polypectomy

In prospective and observational CSP series involving small SNADETs (typically ≤10 mm), delayed bleeding has been rare, and perforation has seldom been reported [10, 12, 42].

In a randomized trial of 130 patients, delayed bleeding after CSP was 1.5%, compared with 6.3% after UEMR; no perforations occurred in either group [36].

However, direct evidence on CSP for SNADETs in patients receiving antithrombotic therapy is limited. Duodenal CSP studies report very low delayed adverse‐event rates in small lesions but no antithrombotic‐specific outcomes [10, 12, 42]. Colorectal evidence is more extensive. Randomized trials and meta‐analyses, mainly involving small or subcentimeter colorectal polyps, have shown lower rates of delayed bleeding with CSP than with hot‐snare or conventional polypectomy in patients receiving antithrombotic agents. Nevertheless, the bleeding risk remains higher than that in patients not receiving periprocedural antithrombotic agents [43, 44]. These findings may favor cold over hot resection when duodenal resection is indicated, but they do not establish the safety of continued antithrombotic therapy during duodenal CSP. Management should therefore follow established periendoscopic guidelines, with decisions regarding continuation or interruption individualized according to thrombotic and bleeding risks [45].

A systematic review and meta‐analysis of cold snare resection, including CSP and CS‐EMR, reported low rates of delayed bleeding (approximately 1%) and infrequent perforation [46]. Systematic reviews and comparative studies have generally shown a lower risk of delayed bleeding with cold than with thermal resection techniques [47, 48, 49]. In a comparative analysis of hot versus cold snare EMR, cold techniques were associated with fewer thermal‐related complications while achieving comparable resectability [48]. A meta‐analysis reported no statistically significant difference in perforation between cold and thermal resection (odds ratio [OR] 0.31, 95% confidence interval [CI] 0.05–2.87) [47].

### 8.2 Cold Forceps Polypectomy

In the multicenter study of 39 lesions ≤6 mm, no adverse events were reported after CFP [37].

### 8.3 Cold Snare‐EMR

The safety profile of CS‐EMR appears less favorable than that of standard CSP, although CS‐EMR has generally been applied to larger lesions. In a meta‐analysis, pooled adverse events included immediate bleeding (4.2%), delayed bleeding (3.4%), and perforation (2.8%) [39]. In a comparative study of large adenomas, CS‐EMR reduced intraprocedural bleeding (2.0% vs. 37%) and postprocedural bleeding (4.0% vs. 16.7%) compared with conventional EMR; however, immediate perforation was observed in 4.0% [38]. A multicenter study reported an overall adverse event rate of 3.9%, including immediate bleeding in 3.1% and delayed bleeding in 0.8%, with no perforations [40].

CS‐EMR may reduce bleeding relative to conventional EMR for large lesions. Nonetheless, the risk of perforation remains a concern and should inform patient selection.

## 9 Surveillance After Cold Resection

### 9.1 Rationale for Surveillance

Cold resection for SNADETs requires structured surveillance because histologic assessment of horizontal margins is frequently indeterminate after duodenal CSP, and local recurrence can occur [12, 42].

Despite these limitations and the shallow resection depth, long‐term local control after CSP for carefully selected diminutive and small SNADETs appears favorable. In a long‐term cohort of 35 patients with a median follow‐up of 48 months, the 12‐month relapse‐free survival rate was 97.1%, and only one recurrence (2.7%) was detected at 12 months [42]. In a prospective pilot trial of 18 patients, one recurrence (5.6%) was detected at the 3‐month follow‐up endoscopy [10].

Nonetheless, recurrence estimates should be interpreted cautiously because follow‐up protocols and outcome definitions vary across studies [10, 42]. Prospective duodenal studies incorporated early endoscopic reassessment into their protocols. The optimal surveillance interval remains uncertain [10, 36].

The ESGE recommends surveillance endoscopy at 3 months after the index treatment and, if no recurrence is detected, again at 1 year; subsequent intervals should be adapted to lesion‐ and resection‐related factors [6]. If residual or recurrent lesions are identified, endoscopic management is often feasible. Recurrences after CSP are typically diminutive and can be managed endoscopically, including with repeat cold resection [42].

Consistent with guideline recommendations and published series, repeat endoscopic therapy—repeat resection or, in selected low‐risk situations, focal ablation—can be considered for small residual or recurrent lesions. Conversely, larger recurrences that are non‐lifting or endoscopically suspicious warrant escalation to thermal resection (EMR/ESD) or surgery, as appropriate [6, 10, 36, 42].

## 10 Technical Tips for Safe and Complete CSP in the Duodenum

**FIGURE 2:**
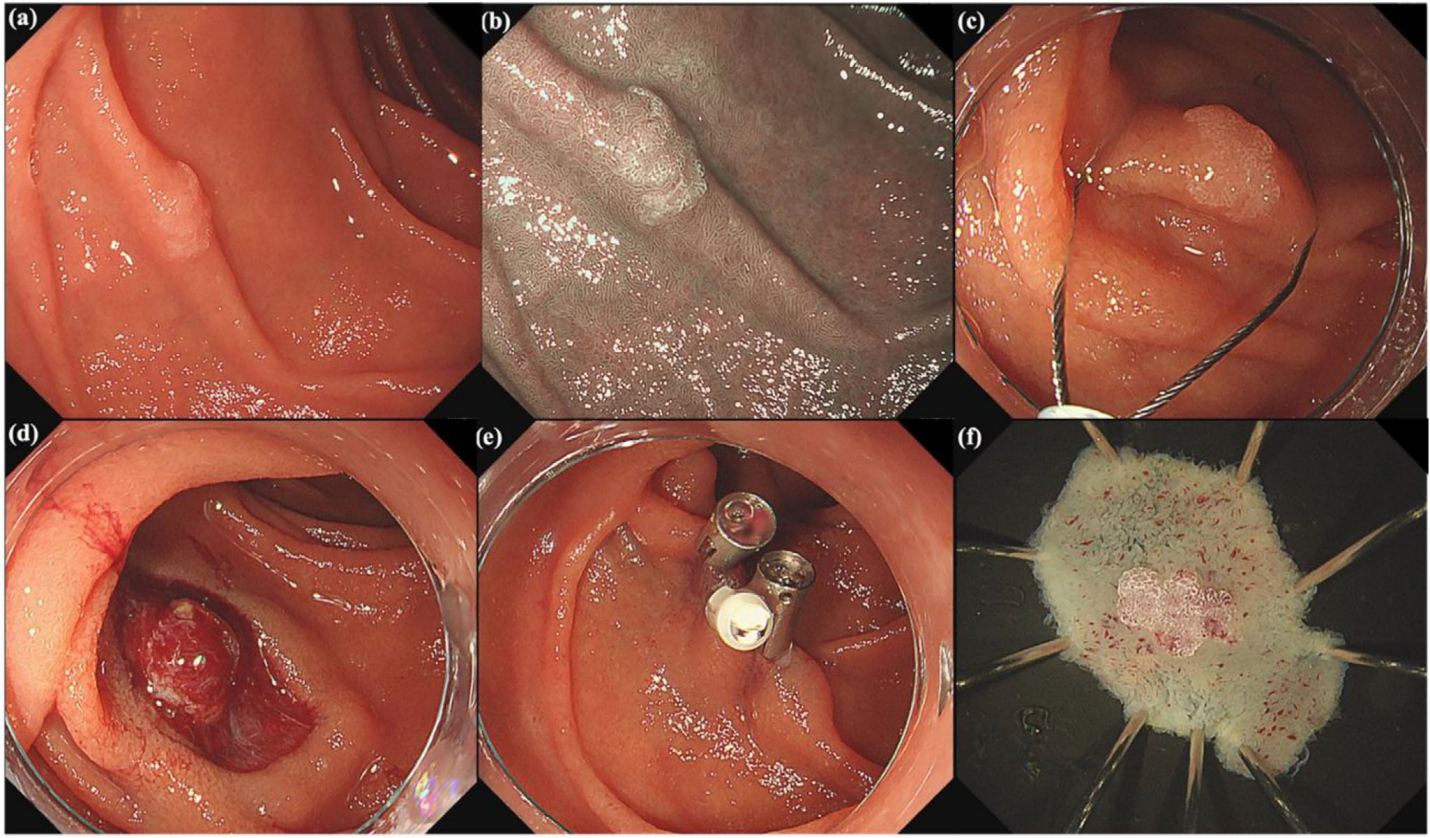
Representative case of cold snare polypectomy (CSP) for a sporadic non‐ampullary duodenal adenoma. (a) White‐light endoscopy shows a 6‐mm, low‐profile, whitish, slightly elevated lesion in the second portion of the duodenum. (b) Narrow‐band imaging shows a clearly demarcated lesion with white opaque substance (WOS). Based on its endoscopic appearance, the lesion was diagnosed as a duodenal adenoma. (c) Cold snare polypectomy was performed with a 10‐mm snare (SnareMaster Plus; Olympus, Tokyo, Japan) without electrocautery, achieving en bloc transection in a single maneuver. (d) Post‐resection endoscopic inspection showed no visible residual lesion at the resection margin. (e) To reduce the risk of delayed bleeding and perforation, prophylactic defect closure was performed with clips (Sure Clip; Micro‐Tech, Nanjing, China; and EZ Clip; Olympus, Tokyo, Japan). (f) The resected specimen measured 9 × 7 mm. Histopathologic examination revealed a 6‐mm intestinal‐type tubular adenoma with negative horizontal and vertical margins.

Figure 2 summarizes key technical steps for safe and complete duodenal CSP, including scope positioning, controlled transection, specimen handling, and defect management.

### 10.1 Stable Positioning and Visualization

Achieve a stable scope position and optimize visualization to allow controlled snare deployment within the narrow, angulated duodenal lumen. When feasible, a non‐tangential approach may improve lesion capture, particularly for flat lesions.

### 10.2 Snare Placement and Controlled Transection

Open the snare fully and, when feasible, include the entire lesion with a small rim of surrounding normal mucosa [36]. Close the snare gradually to achieve controlled mechanical transection [10]. If transection is not achieved smoothly, avoid excessive force and consider switching to an alternative technique or reassessing the indication [6, 36]. Because histologic horizontal margins are frequently indeterminate after duodenal CSP, confirmation of macroscopic clearance and meticulous inspection of the post‐resection site, using chromoendoscopy or image‐enhanced endoscopy when appropriate, are essential [12, 42].

### 10.3 Specimen Retrieval and Handling

The specimen should be retrieved carefully to minimize fragmentation and handling artifacts that may compromise margin assessment [12, 42]. Because CSP specimens often contain limited submucosa, correct orientation and gentle handling remain important for pathologic evaluation [35, 36].

### 10.4 Defect Management

Immediate oozing may occur and can usually be managed mechanically [10, 36]. Selective clip closure should be considered based on endoscopic findings and patient‐related bleeding risk. The defect should be inspected carefully to exclude deep mural injury [35, 36]. The ESGE guideline suggests that defect closure may help mitigate delayed adverse events, although the supporting evidence remains limited [6].

## 11 Conclusion

Management of small low‐grade adenomas should be individualized, with surveillance considered as an alternative to immediate resection. When resection is chosen, CSP is the principal cold resection technique for small (<10 mm) low‐grade adenomas. Careful optical assessment is essential because CSP provides only a shallow resection plane. Other cold resection techniques have more limited roles. Further prospective studies are needed to refine patient selection and surveillance strategies.

## Author Contributions

**Yuichiro Mikuriya**: conceptualization, investigation, data curation, and writing – original draft preparation. **Kohei Takizawa**: conceptualization, methodology, supervision, project administration, and writing – review & editing. **Nobuhisa Minakata**: investigation, validation, and writing – review & editing. **Kazuo Shiotsuki**: investigation, validation, and writing – review & editing. All authors read and approved the final manuscript.

## Funding

The authors have nothing to report.

## Conflicts of Interest

The authors declare no conflicts of interest.

## Ethics Statement

The authors have nothing to report.
